# Supplementary material for: EnzML: multi-label prediction of enzyme classes using InterPro signatures
Source: BMC Bioinformatics. 2012 Apr 25;13:61. doi: 10.1186/1471-2105-13-61 (PMC3483700; doi:10.1186/1471-2105-13-61)
Supplement: Addtional file 5 — The Java code to format the data files, evaluate and predict. The file enzml_java_code.tar.gz contains the Java code used to format database data to ARFF and XML formats, to execute cross and train-test (jackknife) evaluations and to record evaluation results to database. More information is included in the readme.txt file and the Javadoc files. The code can be used with a MySQL database. To use a different database software, other JDBC drivers might be required. [file 1471-2105-13-61-S5.gz › java_code/utils/doc/uk/ac/ed/inf/utils/database/TableCreator.html]

TableCreator


---


|  |  |  |
| --- | --- | --- |
| **Overview** \| **Package** \| **Class** \| **Use** \| **Tree** \| **Deprecated** \| **Index** \| **Help** | |  |
| **PREV CLASS**   **NEXT CLASS** | **FRAMES**    **NO FRAMES**     **All Classes** |  |
| SUMMARY: NESTED \| FIELD \| CONSTR \| METHOD | DETAIL: FIELD \| CONSTR \| METHOD |  |


---


## uk.ac.ed.inf.utils.database Class TableCreator

```
java.lang.Object
  uk.ac.ed.inf.utils.database.DbManaged
      uk.ac.ed.inf.utils.database.TableManaged
          uk.ac.ed.inf.utils.database.TableCreator
```

---

``` public class TableCreator extends TableManaged ```

Class to create a table object in the database. The table data (name etc.) is
taken from a `Table` object.

**Version:**
:   10 Jun 2010

**Author:**
:   Luna De Ferrari luna.deferrari-at-ed.ac.uk

---

**Constructor Summary**

`TableCreator(TableManager tableManager,
Table table)`


| **Method Summary** | |
| --- | --- |
| `boolean` | `createTable()`             Creates the database table for this `Table` object |
| `void` | `dropCreateTable()`             Drops and creates table corresponding to this Table object. |
| `boolean` | `dropTable()`             Drops the database table for this Table object. |
| `java.lang.String` | `getSqlToCreateTable()`             Get the SQL statement to create the table |
| `java.lang.String` | `getTableName()` |

**Methods inherited from class uk.ac.ed.inf.utils.database.TableManaged**

`getTableManager`

**Methods inherited from class uk.ac.ed.inf.utils.database.DbManaged**

`getDbConnection, getDbManager`

**Methods inherited from class java.lang.Object**

`equals, getClass, hashCode, notify, notifyAll, toString, wait, wait, wait`

**Constructor Detail**

### TableCreator

```
public TableCreator(TableManager tableManager,
                    Table table)
```


**Method Detail**

### createTable

```
public boolean createTable()
```

:   Creates the database table for this `Table` object

    :   **Returns:**: true if the table was created, false if the table could not be created or already exists in database

---


### dropCreateTable

```
public void dropCreateTable()
```

:   Drops and creates table corresponding to this Table object. Beware! risk
    of data loss. Better to rename the table and relaunch than dropping it.

    :   **Throws:**: `java.sql.SQLException`

---


### dropTable

```
public boolean dropTable()
```

:   Drops the database table for this Table object. Beware! risk of data
    loss. Better to rename the old table and relaunch than to drop the table.

    :   **Returns:**: true if the table does not exist in the database anymore

---


### getSqlToCreateTable

```
public java.lang.String getSqlToCreateTable()
```

:   Get the SQL statement to create the table

    :   **Returns:**: the create table statement, including all the fields definitions and primary keys

---


### getTableName

```
public java.lang.String getTableName()
```


---


|  |  |  |
| --- | --- | --- |
| **Overview** \| **Package** \| **Class** \| **Use** \| **Tree** \| **Deprecated** \| **Index** \| **Help** | |  |
| **PREV CLASS**   **NEXT CLASS** | **FRAMES**    **NO FRAMES**     **All Classes** |  |
| SUMMARY: NESTED \| FIELD \| CONSTR \| METHOD | DETAIL: FIELD \| CONSTR \| METHOD |  |


---
